# Enhanced Tolerability and Improved Outcomes in Acne Management: A Real‐World Study of Dermocosmetic Adjunctive Therapy

**DOI:** 10.1111/jocd.70372

**Published:** 2025-07-30

**Authors:** Aqsa Siddique, Ahmad Furqan Anjum

**Affiliations:** ^1^ Shaikh Khalifa Bin Zayed Al‐Nahyan Medical College Shaikh Zayed Postgraduate Medical Institute (SZPGMI) Lahore Pakistan

Dear Editor,

We read with great interest the recently published article titled “Enhanced Tolerability and Improved Outcomes in Acne Management: A Real‐World Study of Dermocosmetic Adjunctive Therapy” by Suh et al. [1], which offers a timely and clinically relevant exploration of dermocosmetics as supportive agents in acne treatment. The authors are to be commended for addressing an important and often overlooked aspect of acne management; improving tolerability and adherence, particularly in patients undergoing retinoid therapy. Their work provides meaningful insights into the potential psychosocial and clinical benefits of adjunctive skincare and highlights a growing need for holistic treatment approaches. In particular, the study's pragmatic, real‐world design enhances its external validity, and its focus on improving tolerability and adherence offers valuable insights into patient‐centered care; an often underexplored dimension in acne management. However, the study has the following methodological limitations that warrant consideration.

First, a central methodological limitation of the study lies in its absence of a control group, which precluded randomization and blinding; three interrelated design flaws that significantly undermine internal validity. Since participants were assigned based on pre‐existing acne treatments rather than randomly allocated, this led to imbalances in baseline characteristics, with more severe cases disproportionately placed in the retinoid group. Without a comparator arm receiving standard therapy alone, it becomes impossible to isolate the independent effect of the dermocosmetic regimen from confounding variables such as natural disease progression, placebo effects, or increased adherence due to monitoring. Furthermore, the lack of blinding—both of participants and investigators—introduces performance and detection bias, especially in dermatology where subjective outcomes like burning, itching, and perceived skin quality are highly susceptible to expectancy effects. Thiboutot et al. [2] caution that non‐randomized, open‐label designs tend to overstate dermocosmetic efficacy, underscoring the need for rigorous trial designs. Likewise, Khammari et al. [3] illustrated that including a control group receiving only a retinoid‐based regimen allowed them to directly attribute reduced sensitivity to the dermocosmetic intervention. To enhance methodological rigor in future studies, even if full randomization is not feasible, researchers should consider incorporating matched comparator groups or statistical adjustment for baseline severity, alongside the use of blinded outcome assessors and standardized scoring tools. Second, while the study included three Fitzpatrick skin types (III–V), which adds some diversity, its restriction to a single ethnic population still limits generalizability, which was composed exclusively of Korean participants. This homogeneity restricts the generalizability of the findings to broader, multiethnic populations, particularly those with varying Fitzpatrick skin types (e.g., I, V, VI) who may exhibit different responses to retinoid‐based treatments and dermocosmetics due to inherent differences in skin barrier function, sebaceous activity, and microbiome composition. Ethnic variations have been shown to influence both acne pathophysiology and the tolerability of topical agents, making it inappropriate to extrapolate these results to global populations without caution. Dias da Rocha et al. [4] emphasized this concern in their comprehensive analysis of acne in diverse female populations, highlighting the need for ethnically inclusive studies that account for differing dermatological and hormonal profiles across populations to ensure that treatment recommendations are both effective and equitable. Third, an important but unaddressed limitation of the study is the potential conflict of interest stemming from the involvement of an author affiliated with L'Oréal Korea, the manufacturer of the dermocosmetic products tested. While no conflicts of interest were declared in the manuscript, one author's known affiliation with L'Oréal Korea raises questions about potential undisclosed industry involvement. Even in the absence of explicit financial conflict, such relationships may introduce subconscious bias or influence data interpretation. Although disclosed, such affiliations raise concerns about the possibility of interpretation bias, selective outcome reporting, or overly favorable presentation of results. Industry‐sponsored dermatological research is particularly vulnerable to these biases, especially in trials assessing subjective outcomes such as tolerability and quality of life. While sponsorship does not inherently invalidate findings, it necessitates rigorous safeguards such as third‐party data analysis and independent replication. Blume‐Peytavi et al. [5] cautioned that industry involvement without robust methodological transparency can erode the perceived credibility of skincare research and emphasized the need for independent oversight and publication of both positive and negative results to maintain scientific integrity. Fourth, another methodological limitation of the study is its exclusive reliance on subjective and semi‐quantitative measures—such as investigator‐assessed erythema and patient‐reported symptoms—for evaluating skin sensitivity and treatment response. The absence of objective digital tools or biophysical measurements, such as sebumetry, corneometry, transepidermal water loss (TEWL), or AI‐based imaging, undermines the reproducibility and accuracy of the findings. Dermatological outcomes, especially those involving subtle changes in skin barrier function or sensitivity, benefit significantly from quantifiable instrumentation that reduces inter‐observer variability. Grech et al. [6] emphasized that integrating AI and imaging‐based tools into cosmetology research greatly improves the precision of skin evaluations and helps discern real effects from placebo or subjective bias. Their findings support the necessity of incorporating standardized digital assessments to validate dermocosmetic efficacy more rigorously in clinical research. Given the growing availability and affordability of objective assessment tools—such as TEWL, corneometry, and imaging algorithms—future studies should adopt this as standard practice to enhance reproducibility and minimize observer bias. Fifth, a limitation of the study is its use of a multi‐step dermocosmetic regimen, which included both a cleanser and a moisturizer containing several active ingredients. This layered approach complicates the interpretation of clinical outcomes, as it becomes difficult to distinguish whether the observed benefits are attributable to the moisturizer alone, the combined regimen, or specific ingredients within the formulation. The short contact time of cleansers, in particular, raises questions about the plausibility of meaningful dermal absorption or clinical efficacy from their active components. Furthermore, the DC cream used in the study contains a complex blend of agents—including niacinamide, *Bixa orellana* seed extract, mannose, and Aqua Posae Filiformis (APF); each with distinct biological activities. Without disaggregated analysis, attributing efficacy to any single component is scientifically unsound. As emphasized by Emerald [7], multi‐compound dermatological formulations often lack clarity in mechanistic attribution, and their efficacy cannot be reliably established unless components are evaluated individually or within factorial study designs that can parse out synergistic versus independent effects. For enhanced interpretability, future research should simplify test protocols or structure trials to isolate product components, thereby improving the clinical and mechanistic validity of results.

In conclusion, while the study provides valuable preliminary insights into the adjunctive use of dermocosmetics in acne management, its methodological shortcomings, including the absence of a control group and randomization, limited ethnic diversity, potential industry bias, and reliance on subjective outcome measures, significantly constrain the reliability and generalizability of its findings. These limitations not only hinder causal inference but also pose practical risks when such findings are prematurely translated into clinical recommendations. For example, in the absence of adequate controls, clinicians may mistakenly attribute improvements in skin tolerability to dermocosmetic products, leading to their routine prescription despite limited evidence; an issue avoided in more rigorous studies like that of Khammari et al. [3], where a matched control group helped isolate the true effect of adjunctive treatment.

To enhance the quality of future observational studies, we recommend two immediate corrective strategies: first, the inclusion of well‐matched comparator groups using methods such as propensity score matching or stratified analysis to minimize baseline imbalances; and second, the implementation of blinded outcome assessments, especially for subjective measures such as erythema, sensitivity, and quality of life. These pragmatic enhancements can improve internal validity even outside randomized designs. Long‐term, we continue to advocate for randomized, double‐blind, placebo‐controlled trial designs; inclusion of ethnically and geographically diverse populations; incorporation of independent oversight; and the integration of objective digital or biophysical assessment tools. Collectively, these measures are essential to validate dermocosmetic efficacy and ensure their evidence‐based integration into global acne treatment guidelines.

## Author Contributions

Aqsa Siddique and Ahmad Furqan Anjum conceived the idea, reviewed the original article, performed the literature search, and drafted the manuscript. All authors reviewed and approved the final version of the letter.

## Ethics Statement

The authors have nothing to report.

## Consent

The authors have nothing to report.

## Conflicts of Interest

The authors declare no conflicts of interest.

## Linked Articles

This article is linked to https://doi.org/10.1111/jocd.16772.

## Data Availability

The authors have nothing to report.

## References

[1] D. H. Suh , T. Kim , S. J. Lee , S. J. Jeong , E. Baek , and M. K. Shin , “Enhanced Tolerability and Improved Outcomes in Acne Management: A Real‐World Study of Dermocosmetic Adjunctive Therapy,” Journal of Cosmetic Dermatology 24, no. 1 (2025): e16772. 39757973 10.1111/jocd.16772 PMC11701793

[2] D. Thiboutot , A. M. Layton , I. Traore , et al., “International Expert Consensus Recommendations for the Use of Dermocosmetics in Acne,” Journal of the European Academy of Dermatology and Venereology 39, no. 5 (2025): 952–966. 38877766 10.1111/jdv.20145 PMC12023719

[3] A. Khammari , D. Kerob , A. L. Demessant , M. Nioré , and B. Dréno , “A Dermocosmetic Regimen Is Able to Mitigate Skin Sensitivity Induced by a Retinoid‐Based Fixed Combination Treatment for Acne: Results of a Randomized Clinical Trial,” Journal of Cosmetic Dermatology 23, no. 4 (2024): 1313–1319. 38102855 10.1111/jocd.16120

[4] M. A. Dias Da Rocha , “Saint Aroman M, Mengeaud V, Carballido F, Doat G, Coutinho A, Et al. Unveiling the Nuances of Adult Female Acne: A Comprehensive Exploration of Epidemiology, Treatment Modalities, Dermocosmetics, and the Menopausal Influence,” International Journal of Women's Health 16 (2024): 663–678. 10.2147/IJWH.S431523 PMC11034510 38650835

[5] U. Blume‐Peytavi , M. Bagot , D. Tennstedt , et al., “Dermatology Today and Tomorrow: From Symptom Control to Targeted Therapy,” Journal of the European Academy of Dermatology and Venereology 33, no. S1 (2019): 3–36. 10.1111/jdv.15335 30561009

[6] V. S. Grech , V. Kefala , and E. Rallis , “Cosmetology in the Era of Artificial Intelligence,” Cosmetics 11, no. 4 (2024): 135.

[7] M. Emerald , “Medicinal Plants: Therapeutic Potential, Safety, and Toxicity,” in Drug Discovery and Evaluation: Safety and Pharmacokinetic Assays (Springer, 2024), 1327–1397, https://link.springer.com/rwe/10.1007/978‐3‐031‐35529‐5_90.

